# Altered Brain Activity during Reward Anticipation in Pathological Gambling and Obsessive-Compulsive Disorder

**DOI:** 10.1371/journal.pone.0045938

**Published:** 2012-09-20

**Authors:** Jung-Seok Choi, Young-Chul Shin, Wi Hoon Jung, Joon Hwan Jang, Do-Hyung Kang, Chi-Hoon Choi, Sam-Wook Choi, Jun-Young Lee, Jae Yeon Hwang, Jun Soo Kwon

**Affiliations:** 1 Department of Psychiatry, Seoul National University College of Medicine, Seoul, Korea; 2 Department of Psychiatry, SMG-SNU Boramae Medical Center, Seoul, Korea; 3 Department of Psychiatry, Kangbuk Samsung Hospital, Sungkyunkwan University School of Medicine, Seoul, Korea; 4 Institute of Human Behavioral Medicine, SNU-MRC, Seoul, Korea; 5 Department of Radiology, National Medical Center, Seoul, Korea; 6 Department of Addiction Rehabilitation and Social Welfare, Eulji University, Seongnam, Korea; 7 Brain & Cognitive Science-WCU program, College of Natural Science, SNU, Seoul, Korea; Institute of Psychiatry at the Federal University of Rio de Janeiro, Brazil

## Abstract

**Background:**

Pathological gambling (PG) and obsessive-compulsive disorder (OCD) are conceptualized as a behavioral addiction, with a dependency on repetitive gambling behavior and rewarding effects following compulsive behavior, respectively. However, no neuroimaging studies to date have examined reward circuitry during the anticipation phase of reward in PG compared with in OCD while considering repetitive gambling and compulsion as addictive behaviors.

**Methods/Principal Findings:**

To elucidate the neural activities specific to the anticipation phase of reward, we performed event-related functional magnetic resonance imaging (fMRI) in young adults with PG and compared them with those in patients with OCD and healthy controls. Fifteen male patients with PG, 13 patients with OCD, and 15 healthy controls, group-matched for age, gender, and IQ, participated in a monetary incentive delay task during fMRI scanning. Neural activation in the ventromedial caudate nucleus during anticipation of both gain and loss decreased in patients with PG compared with that in patients with OCD and healthy controls. Additionally, reduced activation in the anterior insula during anticipation of loss was observed in patients with PG compared with that in patients with OCD which was intermediate between that in OCD and healthy controls (healthy controls < PG < OCD), and a significant positive correlation between activity in the anterior insula and South Oaks Gambling Screen score was found in patients with PG.

**Conclusions:**

Decreased neural activity in the ventromedial caudate nucleus during anticipation may be a specific neurobiological feature for the pathophysiology of PG, distinguishing it from OCD and healthy controls. Correlation of anterior insular activity during loss anticipation with PG symptoms suggests that patients with PG fit the features of OCD associated with harm avoidance as PG symptoms deteriorate. Our findings have identified functional disparities and similarities between patients with PG and OCD related to the neural responses associated with reward anticipation.

## Introduction

Pathological gambling (PG) is a chronic disorder that occurs primarily in men and is characterized by a persistent pattern of continued gambling behavior despite adverse consequences. PG is considered a behavioral addiction [1], with a dependency on repetitive gambling behavior. Obsessive-compulsive disorder (OCD) has also been conceptualized as a behavioral addiction [2], [3], and compulsion in OCD could be viewed as addictive because there are rewarding effects following reduction of anxiety induced by obsession [4].

Addictive behaviors in PG and OCD are associated with dysfunctional process of the reward system. The reward processing is dependent on ventral striatal-orbitofrontal circuitry in brain [5]. However, there is evidence of a functional dissociation between reward anticipation and outcome rather than a unitary reward circuit [6]. In healthy subjects, the ventral striatum is activated during anticipation and the anterior insula is activated especially during loss anticipation, whereas the prefrontal region, including the medial prefrontal and orbitofrontal cortex (OFC), shows greater activation in response to actual rewards [6], [7]. With respect to defective reward processing in substance addiction, Beck et al. [8] reported that male alcoholics show reduced activation of the ventral striatum during the anticipation of monetary gain relative to that in healthy controls. Cocaine abusers demonstrated reduced regional brain activation in the corticolimbic reward circuit, including the OFC, in response to monetary reward gradations [9]. On the other hand, patients with OCD showed decreased reward anticipation activity in the nucleus accumbens, part of the ventral striatum [4]. In a previous report, our group revealed increased activation of the anterior insula during the anticipation of monetary loss in patients with OCD relative to that in healthy controls, suggestive of elevated sensitivity to negative cues in OCD [10]. Several studies in patients with PG have found abnormalities in the reward systems of these individuals. Reuter et al. [11] reported reduced activation of the fronto-striatal circuit in response to monetary rewards, suggesting that PG is characterized by a blunted response to reward stimuli similar to that found in substance addiction. Recently, de Ruiter et al. [12] reported that PG is related to response perseveration and diminished reward and punishment sensitivity, as indicated by hypoactivation of the ventrolateral prefrontal cortex when money was gained or lost. While these studies looked at outcome of reward, Balodis et al. [13] found decreased activity in the ventral striatum during reward anticipation and in the anterior insula during loss anticipation.

Considering repetitive gambling and compulsion as addictive behaviors, investigation of neurobiological features in PG and OCD may be helpful for understanding addictive behaviors. To date, however, no neuroimaging studies have investigated reward circuitry in PG compared with in OCD. Brain activity during the anticipation of reward may be important as it might influence subsequent choices and behaviors. Therefore, we applied the monetary incentive delay task [14] in the context of event-related functional magnetic resonance imaging (fMRI) to elucidate functional brain activity specific to the anticipation phase of a reward condition among patients with PG and compared the results with those of patients with OCD and healthy controls. In this study, based on the previous reports indicating diminished positive reward and punishment sensitivity to reward in PG, we hypothesized that patients with PG would show decreased neural activities in the ventral striatum during the anticipation phase of both gain and loss relative to those in healthy controls, and decreased activities in PG would be comparable to those in OCD. In addition, we expect to find decreased anterior insular activities in patients with PG during the anticipation phase of loss relative to those in patients with OCD and healthy controls.

We included only male subjects in this study based on the findings showing predominant male prevalence in PG and sex differences in the neural response to reward stimuli [15]–[17]. We also included young adult patients with PG who had an illness duration of <5 years to elicit earlier brain changes in the reward system of PG.

## Materials and Methods

### Ethics statement

This study was conducted according to the principles expressed in the Declaration of Helsinki. The institutional review boards of the Kangbuk Samsung Medical Center and Seoul National University Hospital approved the study protocol, and all subjects signed an informed consent form prior to participation.

### Participants

Fifteen male patients with PG (age, 27.93±3.59 years), 15 age-and IQ-matched healthy male controls (age, 26.60±4.29 years), and 13 male patients with OCD (age, 24.92±6.92 years) were enrolled. Previously, our group reported fMRI study on neural activities in reward system of OCD [10]. In the current study, only male subjects with OCD were derived from our previous report [10] for sex matching to those with PG and healthy controls. Patients were recruited from the outpatient clinics of Kangbuk Samsung Hospital and Seoul National University Hospital, Seoul, South Korea and diagnoses of PG or OCD were based on the Structured Clinical Interview for DSM-IV (SCID) [18]. The diagnosis of PG was also defined for patients with a South Oaks Gambling Screen (SOGS) score ≥5 [19]. No significant difference in education level was observed among the three groups. All subjects were right-handed except one in the PG group. Clinical assessments included the Yale-Brown Obsessive Compulsive Scale for pathological gambling (PG-YBOCS) [20] to measure PG symptom severity, the YBOCS for measuring the severity of obsessive compulsive symptoms, the Beck Depression Inventory (BDI) [21] for depressive symptoms, and the Beck Anxiety Inventory (BAI) [22] for anxiety symptoms. BDI scores in both PG and OCD were above 15, but no patients were diagnosed with major depressive disorder by SCID. In the OCD group, 10 patients were free from comorbidity and the following disorders were comorbid: tic disorder (N = 1), obsessive-compulsive personality disorder (N = 1), and schizotypal personality disorder (N = 1). In the PG group, no patients had comorbidities. In addition, patients with OCD were classified into the following subtypes: contamination/cleaning (N = 5), aggression/checking (N = 6), sexual/religious (N = 1), and symmetry/ordering (N = 1). No patient with OCD had hoarding subtype. The status of smoking was as follows: PG (N = 7), OCD (N = 6), and control (N = 8). There was no significant difference in the status of smoking among groups.

Healthy controls were recruited from the local community and had no history of any psychiatric disorder. Exclusion criteria for all subjects were a history of significant head injury, alcohol or substance abuse, seizure disorder, and psychotic disorder.

All participants were medication naïve at the time of assessment. The Korean version of the Wechsler Adult Intelligence Scale was administered to all subjects to estimate IQ.

### Experimental paradigm

We used the monetary incentive task developed by Knutson et al. [14]. The task used in this study was described in our previous report [10]. During each trial, subjects viewed one of three cues (cue duration, 350 ms) indicating that their response would result in winning money, avoiding monetary loss, or no monetary outcome. After the cue, participants fixated on a crosshair as they waited for a variable interval (delay duration, 4180–4480 ms) before pressing a button in response to a white target square that appeared for a variable length of time (target duration, 200–500 ms). Feedback (i.e., the outcome, 1500 ms in duration) following the disappearance of the target notified participants whether they had won or lost money during the preceding trial and presented their winnings.

Cues indicating potential gain, potential loss, and no monetary outcome are denoted by red, blue, and yellow circles, respectively. The amount of money at stake in each trial was 1000 Korean Won (approximately 1 US dollar). Three cues were pseudo-randomly presented with a fixation crosshair, separated by a jittered inter-trial interval of 5.17–9.85 s. Before MRI scanning, all participants completed an unpaid short practice session for each cue to minimize learning effects and provide an estimate of each individual's reaction time (RT) to standardize task difficulty. Mean RT during the practice session was used as duration of the target exposure in the first trial during MRI scanning. The target durations decreased by 30 ms on the next trial after a correct response and increased by 40 ms on the next trial after an incorrect response. As result, success rates in PG, OCD and healthy control were 57.27%, 54.48%, and 58.08%, respectively (Table 1). Participants received their cumulative total amount of the money won during the task performances.

**Table 1:** Demographic and clinical characteristics of the subjects a.

Variables	Control (N = 15)	PG (N = 15)	OCD (N = 13)	F	p
**Demographic data**					
Age (years)	26.60 (4.29)	27.93 (3.59)	24.92 (6.92)	1.25	0.30
Education (years)	14.27 (1.39)	14.80 (1.70)	14.38 (3.07)	0.26	077
IQ score	114.47 (7.10)	113.67 (9.96)	108.54 (14.59)	1.21	0.31
**Clinical data**					
Age of Onset (years)		25.67 (3.92)	16.23 (5.66)	26.89	<0.01^b^
Duration of illness (years)		2.20 (1.29)	8.69 (5.68)	18.62	<0.01^b^
YBOCS c					
* Total score*		16.13 (7.28)	19.54 (5.94)	1.80	0.19
* Obsession score*		8.40 (3.46)	10.31 (4.68)	1.53	0.23
* Compulsion score*		7.73 (4.01)	9.23 (4.55)	0.86	0.36
SOGS		15.90 (1.73)			
BDI	3.00 (4.81)	15.20 (12.32)	15.08 (10.42)	7.68	<0.01^b^
BAI	3.67 (3.85)	11.00 (13.40)	14.08 (16.09)	2.80	0.07
Smoking (cigarettes a day)	9.33 (9.61)	8.67 (9.90)	8.46 (9.87)	0.03	0.97
**Behavioral data**					
Total earnings (in Korean Won)	28000.00 (2360.39)	27266.67 (2120.20)	28923.08 (3226.49)	1.44	0.25
Reaction times (ms overall)	222.51 (19.40)	231.33 (26.26)	215.48 (23.60)	1.64	0.21
Hit rate (% overall)	58.08 (1.36)	57.27 (1.88)	58.48 (1.71)	1.93	0.16

Data are given as mean (SD).

PG, Pathological Gambling; OCD, Obsessive-compulsive disorder; IQ, Intelligence Quotient; BDI, Beck Depression Inventory; BAI, Beck Anxiety Inventory; YBOCS, Yale-Brown Obsessive Compulsive Scale; SOGS, South Oaks Gambling Screen.

a One-way analysis of variance (ANOVA) was used.

b *p*<.05.

c YBOCS was administered for patients with OCD and PG-YBOCS was used for patients with PG.

### Image acquisition

MR scanning was performed on a 1.5 Tesla Scanner (Siemens, AVANTO, Munich, Germany). Twenty-five axial slices were obtained using a gradient-echo echo planar imaging (EPI) sequence (TE/TR, 52/2340 ms; FOV, 220×220 mm^2^; FA, 90°, 3.44×3.44×5 mm^3^, interleaved, no interslice gap); we collected an interleaved slice-acquisition pattern, approximately parallel to the anterior-posterior commissure plane to avoid signal contamination from adjacent slices. Functional images were obtained in two runs of 538.2 s each, resulting in 245 volumes per run for each subject. High resolution T1-weighted structural MRI images (MPRAGE, TR, 11.6 s; TE, 4.76 ms; FOV, 230 mm; FA, 15°; 208 slices, 0.45×0.45×0.9 mm^3^) were also acquired for each subject for anatomical reference. Stimuli were back-projected onto a translucent screen located at the subject's feet using an LCD projector and viewed through a periscope mirror attached to the head coil. The subjects responded to the target by pressing an MRI-compatible mouse button with the index finger of the right hand.

### Functional MRI processing and statistical analysis

Data were analyzed using Statistical Parametric Mapping software (SPM8, Wellcome Department of Imaging Neuroscience, London, UK). The first three volumes of EPI images were discarded due to magnetization instability. The EPI images were corrected for differences in acquisition time, realigned, spatially normalized to the Montreal Neurology Institute reference brain (voxel size 3×3×3 mm^3^), smoothed using an isotropic 4-mm full-width half-maximum Gaussian kernel, and high-pass filtered with a 128-s cut-off. After images were preprocessed, the first-level analysis was performed on each subject by modeling the different conditions (cues indicating potential gain, potential loss, and no outcome) and convolved with a canonical hemodynamic response function according to the general linear model. Movement parameters were also included in the statistical model. The relevant contrasts were as follows: gain cue vs. no-outcome cue to assess the main effect of gain anticipation, and loss cue vs. no-outcome cue to assess the main effect of loss anticipation. A within-group analysis and a between-group analysis were performed for the contrast images for gain and loss anticipation. The results of the between-group activation are presented in Tables 2 and 3. We used a threshold of *P*<0.05, family-wise error (FWE)-corrected to verify the activation clusters of the mesolimbic reward system (e.g., the ventral striatum) for whole brain analyses in each group. Significant differences in the between-group analysis were reported using the criteria of *P*<0.001, uncorrected, cluster size ≥7 (189 µl), which corresponds to a corrected threshold of *P*<0.05 as determined by AlphaSim in AFNI (http://afni.nih.gov/afni/docpdf/AlphaSim.pdf). Our previous report also described this threshold in order to prevent detection of false positives [10].

**Table 2:** Brain regions showing significant difference of activation during anticipation of gain versus no gain among pathological gambling (PG), obsessive-compulsive disorder (OCD) and healthy controls.

Regions	L/R	MNI coordinates			T/Z-value	voxels
		x	y	Z		
***Healthy controls > PG***						
Thalamus	R	18	−24	0	4.41/3.96	24
Fusiform	R	39	−72	−18	4.56/4.07	16
Middle temporal gyrus	R	57	−51	−12	3.59/3.32	8
Inferior temporal gyrus	R	60	−60	−6	3.97/3.62	21
Middle occipital gyrus	R	39	−81	12	3.94/3/60	39
Lingual gyrus	L	−15	−78	−12	4.01/3.65	18
	L	−21	−60	−9	3.80/3.49	7
Cerebellum	L	−9	−33	−12	3.90/3.57	16
***PG > Healthy controls***						
none						
***Healthy controls > OCD***						
none						
***OCD > Healthy controls***						
none						
***OCD > PG***						
Ventromedial caudate nucleus		0	6	−6	4.81/4.25	11
Caudate	L	−12	27	15	4.73/4.19	27
	R	12	24	9	3.98/3.63	18
Thalamus	R	6	−24	0	3.98/3.63	14
Posterior insula	L	−33	−36	24	5.03/4.40	8
Middle frontal gyrus	L	−21	24	24	5.67/4.83	8
Superior occipital gyrus	R	18	−90	24	4.50/4.02	34
Lingual gyrus	L	−21	−60	−6	4.71/4.18	18
	R	21	−72	0	4.55/4.06	21
***PG > OCD***						
none						

All results *P*<0.001 uncorrected and k≥7 voxels, which corresponds to a corrected threshold *P*<0.05.

**Table 3:** Brain regions showing significant difference of activation during anticipation of loss versus no loss among pathological gambling (PG), obsessive-compulsive disorder (OCD) and healthy controls.

Regions	L/R	MNI coordinates			T/Z-value	voxels
		x	y	z		
***Healthy controls > PG***						
Ventomedial caudate nucleus		0	3	0	4.33/3.90	8
Temporal pole	L	−39	3	−24	4.32/3.89	7
***PG > Healthy controls***						
Rolandic operculum	L	−42	−6	15	4.30/3.88	16
***Healthy controls > OCD***						
none						
***OCD > Healthy controls***						
Anterior insula	L	−18	30	9	4.26/3.85	10
Caudate	L	−15	15	24	4.71/4.17	11
Putamen	R	30	−6	21	4.82/4.25	8
***OCD > PG***						
Ventromedial caudate nucleus		0	3	0	6.24/5.18	25
Caudate	R	12	15	18	4.80/4.24	9
***PG > OCD***						
Inferior parietal gyrus	L	−54	−30	39	4.11/3.73	15
Superior frontal gyrus	L	−18	12	69	3.71/3.42	7

All results *P*<0.001 uncorrected and k≥7 voxels, which corresponds to a corrected threshold *P*<0.05.

In order to test our main hypotheses, we selected regions of interest (ROIs, 5-mm radius spheres) in the ventromedial caudate nucleus (part of the ventral striatum) and the anterior insula and calculated individual mean contrast values for each ROI using Marsbar software (http://marsbar.sourceforge.net). We explored correlations among clinical variables and ROI mean contrast values using Pearson's correlations.

Comparisons of demographic and clinical variables among patients with PG, patients with OCD, and healthy controls were conducted using an ANOVA. Statistical analysis was two-tailed, and significance was set at *P*<0.05.

## Results

### Demographic, clinical and behavioral data

No significant differences in age, education, IQ or BAI score were observed among the three groups (Table 1). However, patients with PG and those with OCD showed higher scores on the BDI than did the control subjects. Patients with OCD showed a longer duration of illness and earlier onset than did patients with PG (*F* = 18.62, *P*<0.01; *F* = 26.89, *P*<0.01, respectively).

With regard to behavioral data, no significant differences were observed among the three groups for the mean hit rate (*F* = 1.93, *P* = 0.16), the mean RT (*F = 1.64*, *P* = 0.21), or the amount of money gained (*F = 1.44*, *P* = 0.25) (Table 1).

### Brain activation

During anticipation of potential gain, all groups showed significant activation in the ventral striatum, frontal cortex and occipital cortex. The extent of activation in the ventral striatum of patients with PG was less than those in healthy controls and patients with OCD. During anticipation of loss, no significant activation was observed in the ventral striatum in the PG group.

**Figure 1:**
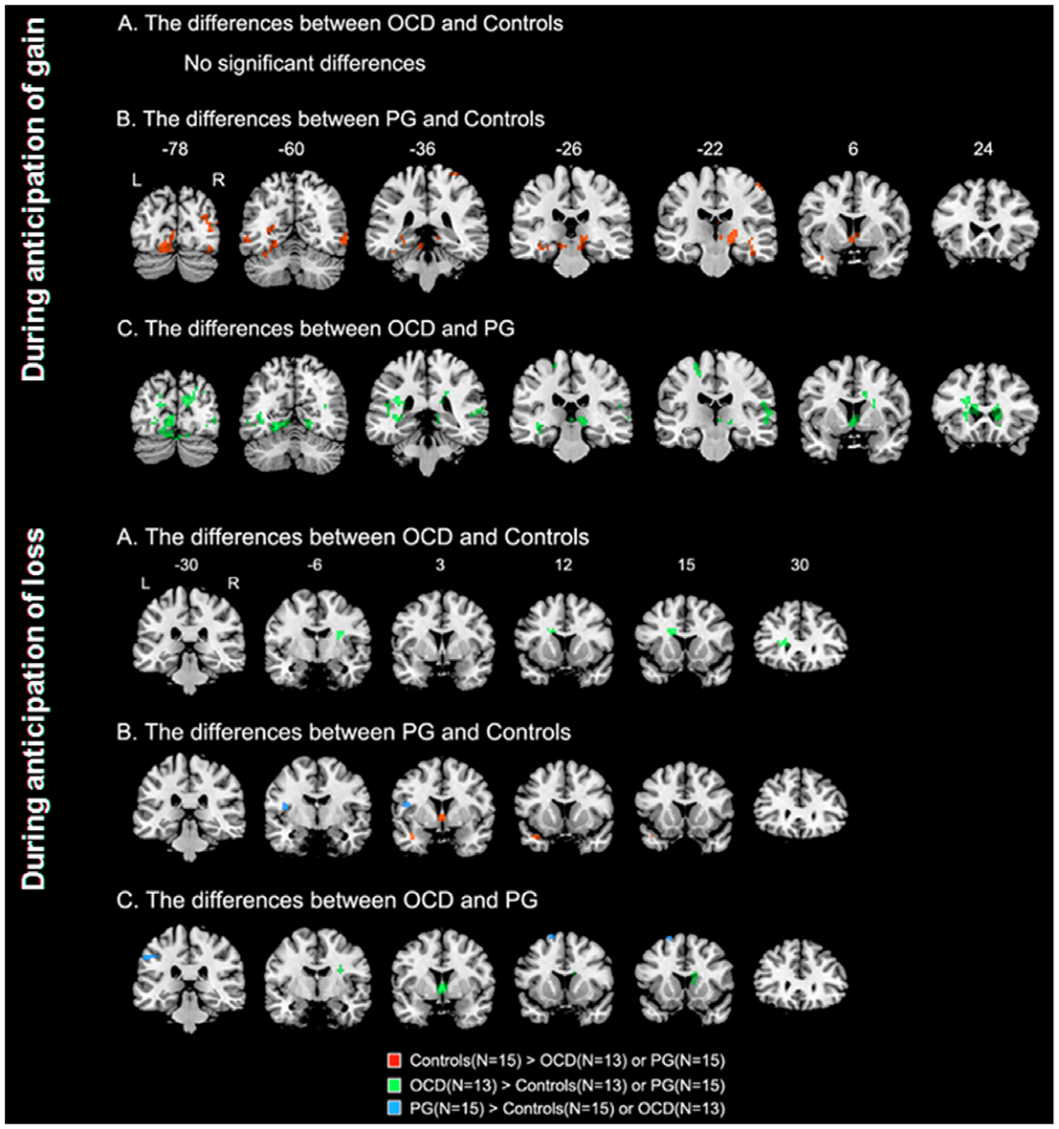
Group differences in whole brain analysis among healthy controls, PG and OCD in response to anticipation of gain and anticipation of loss (for illustrative purpose, *P*<0.005 uncorrected, cluster level 15). PG: Pathological Gambling; OCD, Obsessive-compulsive disorder; L, left; R, right.

**Figure 2:**
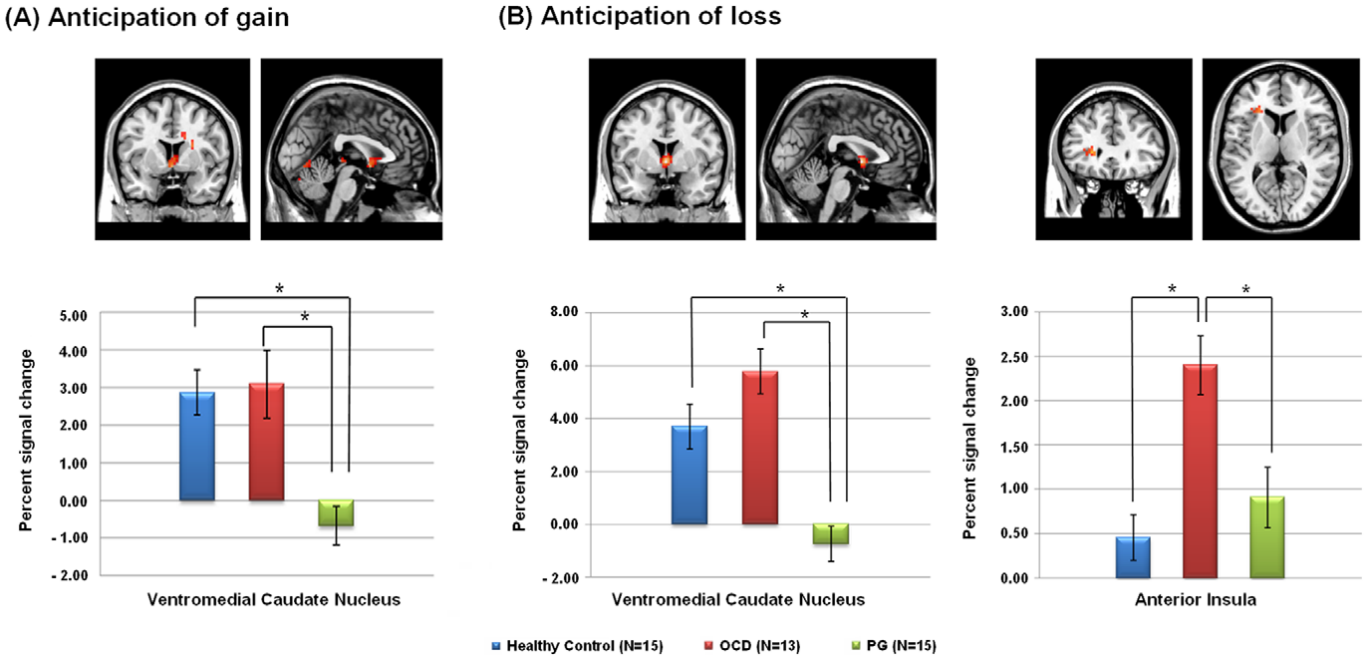
Group differences in percent signal changes of the selected brain regions during the phase of anticipation among healthy controls, pathological gambling (PG) and obsessive-compulsive disorder (OCD). Significant differences in brain activations between groups are marked (asterisk).

In the between-group analysis, patients with PG showed less activation in the ventromedial part of the caudate nucleus as well as middle frontal gyrus, and occipital cortex than did patients with OCD during anticipation of gain. Patients with PG showed reduced activation in the thalamus relative to patients with OCD and healthy controls. No statistical differences in the BOLD signal were observed between patients with OCD and healthy controls during anticipation of gain (Fig. 1). We performed ROI analyses in the ventromedial part of the caudate nucleus to confirm differences in activation. We found significant between-group differences among the three groups for the percent signal change in the ventromedial caudate nucleus (*F*_2, 40_ = 10.02, *P*<0.001). A post hoc analysis revealed that activation in patients with PG decreased significantly in the ventromedial caudate nucleus relative to patients with OCD and healthy controls. However, no significant difference was observed between patients with OCD and healthy controls in this region (Fig. 2).

During anticipation of loss, patients with PG showed significantly less activation in the ventromedial caudate nucleus relative to that in patients with OCD and healthy controls. In contrast, increased activation in the anterior insula as well as in the putamen and caudate nucleus was found in patients with OCD relative to healthy controls (Fig. 1). The ROI analysis showed significant between-group differences among the three groups for the percent signal change in the ventromedial caudate nucleus and anterior insula (*F*_2, 40_ = 17.49, *P*<0.001 in the ventromedial caudate nucleus; *F*_2, 40_ = 10.19, *P*<0.001 in the anterior insula). A post hoc analysis also revealed that ventromedial caudate nucleus activation decreased significantly in patients with PG relative to that in patients with OCD and healthy controls. Patients with OCD showed increased activation in the anterior insula relative to that in patients with PG and healthy controls. The percent signal change in the anterior insula of the PG group was intermediate between that in the OCD and healthy control groups (Fig. 2).

### Correlation between behavioral/clinical variables and brain activation

**Figure 3:**
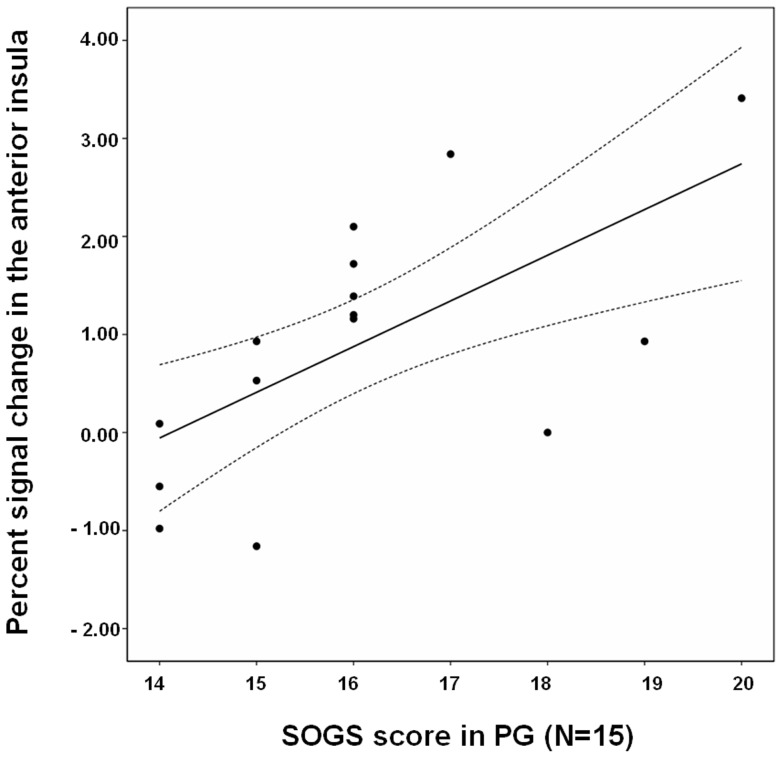
The relationship between brain activity in the anterior insula and South Oaks Gambling Screen (SOGS) score in patients with pathological gambling (PG) during the phase of loss anticipation (r = 0.64, p = 0.02).

We found significant positive correlations between activity in the anterior insula and SOGS score in patients with PG during the anticipation of loss (r = 0.64, *P* = 0.02) (Fig. 3).

## Discussion

To our knowledge, this is the first event-related fMRI study to elucidate neural changes associated with reward anticipation in patients with PG distinguished from those with OCD and healthy controls. We found that activation in the ventromedial caudate nucleus during anticipation of both gain and loss decreased in patients with PG compared with that in patients with OCD and healthy controls. Additionally, reduced activation in the anterior insula during anticipation of loss was observed in patients with PG compared with that in patients with OCD, and a significant positive correlation between activity in the anterior insula and SOGS score was found in patients with PG.

As we expected, we found reduced activation of the ventromedial caudate nucleus during both anticipation of gain and anticipation of loss in patients with PG compared with that in healthy controls. The ventromedial caudate nucleus has been considered a part of the ventral striatum [23], [24], which is strongly innervated by dopaminergic fibers. Dopamine is implicated in reward and reinforcing behavior. The dopaminergic mesolimbic circuit, which may be central to the pathophysiology of addiction [25], receives dopaminergic projections from midbrain neurons, particularly the ventral tegmental area, including both subcortical regions such as the lateral hypothalamus and ventral striatum and cortical regions such as the medial prefrontal cortex [26].

There was an evidence for involvement of the ventral striatum in PG. Patients with PG showed less activation of the ventral striatum during simulated gambling [11]. However, reward anticipation and outcome may differentially recruit distinct brain regions. That is, anticipation of reward activates ventral striatum, whereas outcome of reward activates the ventromedial frontal cortex [6], [27], [28]. Recently, patients with PG exhibited reduced activity in the ventral striatum during reward anticipation [13]. A previous PET study reported that release of dopamine in the ventral striatum is related to the expectation of reward, not to the reward itself [29]. Anticipation represents a critical phase of incentive processing because it has the potential to influence subsequent thoughts and behaviors [30]. Therefore, a hypoactive ventral striatum including the ventromedial caudate nucleus during anticipation of reward in patients with PG may reflect a hypo-dopaminergic state and decreased reward sensitivity, suggestive of high risk for addictive behavior. In contrast, van Holst et al. [31] found increased reward expectancy coding in the striatum of PG relative to controls. Van Holst et al. [31] used a guessing card task during fMRI scanning, which was different from that used in the current and Balodis et al.[13]'s studies in that it has a tendency to induce cue-related brain activity. For example, patients with online gaming addiction showed increased activations in the frontal cortex, nucleus accumbens, and caudate nucleus when viewing gaming pictures, relative to normal controls [32]. Further studies with larger sample sizes and relevant tasks for investigating pure reward system are needed in order to confirm these different findings.

Contrary to our expectations, neural activity in the ventral striatum of PG was less than that in OCD, and patients with OCD and healthy controls showed comparable activation of the ventral striatum during anticipation of gain. It could be speculated that the OCD group in our study was heterogeneous in terms of symptom dimension. Figee et al. [4] reported decreased reward anticipation activity in the nucleus accumbens in OCD, and its decreased activity was more pronounced in patients with OCD with contamination fear than in those with high-risk assessment. In addition, compared with the report by Figee et al. [4], the OCD group in our study had shorter duration of illness as well as less severe symptoms and had only male subjects. These clinical characteristics could have influenced the current results.

Decreased activation of the anterior insula during anticipation of loss was observed in patients with PG relative to that in patients with OCD. The anterior insular activity in patients with PG was intermediate between that in patients with OCD and healthy controls, although anterior insular activity in patients with PG was not significantly different from that in healthy controls. The insula is an important region for emotion processing and its activation is associated with anticipation of adverse events [33]. Moreover, Preuschoff et al. [34] recently showed that activation of the anterior insula reflects both risk prediction and risk prediction errors. Our previous report [10] showed increased activation in the anterior insula of patients with OCD relative to that in healthy controls during anticipation of loss. The current findings in male subjects were almost similar to previous results reported by our group [10]. This may be, in part, associated with the small numbers of female subjects (n = 7) relative to male subjects (n = 13) in the previous report. These findings suggest that patients with OCD may be more responsive to anticipation of loss, resulting in harm avoidance behavior. In contrast, patients with PG showed decreased insular activation relative to that in patients with OCD. However, we found a significant positive correlation between activation of the anterior insula and SOGS score in patients with PG during anticipation of loss. This suggests that young adult patients with PG are more sensitive to anticipation of loss, as their symptoms deteriorate. This is not consistent with a previous report, which showed diminished sensitivity to punishment in patients with PG [12]. The previous study included patients with PG who were older than those in the present study, and the neural activities in the report by de Ruiter et al. [12] were associated with reward outcome. Thus, as the illness progresses, younger patients with PG seem to evidence characteristics more similar to those of OCD, as they are hypersensitive to anticipation of loss. In addition, the findings that young patients with PG are sensitive to anticipation of loss may be related to increased anxiety and depressive symptoms in patients with PG, as in those with OCD. These mood states may contribute to the harm avoidance [35] associated with hypersensitive to anticipation of loss in both patients with PG and those with OCD.

In the present study, a reduction in the thalamic activation was also found in patients with PG during anticipation of gain compared with those with OCD and healthy controls. The thalamus acts as an intermediary between the ventral striatum and medial prefrontal cortex [36], [37]. The thalamus and the ventral striatum are innervated by dopaminergic neurons [38], [39] and have been implicated in reward processing [38]. Dysfunctional connectivity of the mesolimbic circuit may be implicated in patients with PG, considering the important role of the thalamus in the reward circuit.

We found no significant correlations of neural responses in the ventral striatum with clinical variables including symptom severity, duration of illness, and age of onset. Furthermore, patients with PG were drug naïve and were in the early phase after disease onset, i.e., within 5 years after onset: therefore, reduced neural activities in the ventral striatum during anticipation of reward could be trait markers for the pathophysiology of PG, distinguishing it from OCD and healthy controls.

Our study had some limitations. The small sample size and the inclusion of only male participants may limit the generalizability of the results. This limitation was inevitable because our goal was to recruit a homogeneous sample to control for confounding factors such as medication and gender effects. Thus, we recruited patients with PG who had an illness duration of <5 years and were young adults in order to elicit earlier brain changes in the reward system of PG. Furthermore, we selected drug naïve patients with PG.

In summary, our results showed that neural activities in the ventromedial caudate nucleus during anticipation of both gain and loss decreased in patients with PG relative to those in patients with OCD and healthy controls, suggesting that this neural activity may be a trait marker for the pathophysiology of PG. During the anticipation of loss, anterior insular activation in patients with PG was intermediate between that in patients with OCD and healthy controls and was significantly correlated with PG symptom severity, suggesting that patients with PG fit the features of OCD as symptoms deteriorate. Our findings have identified functional disparities and similarities between patients with PG and OCD related to the neural responses associated with reward anticipation.
